# Feature tracking microfluidic analysis reveals differential roles of viscosity and friction in sickle cell blood†

**DOI:** 10.1039/d1lc01133b

**Published:** 2022-03-16

**Authors:** Hannah M. Szafraniec, José M. Valdez, Elizabeth Iffrig, Wilbur A. Lam, John M. Higgins, Philip Pearce, David K. Wood

**Affiliations:** Department of Biomedical Engineering, University of Minnesota Minneapolis Minnesota USA dkwood@umn.edu; Department of Medicine, Emory University Atlanta Georgia USA; Aflac Cancer Center and Blood Disorders Service of Children's Healthcare of Atlanta Atlanta Georgia USA; Department of Mathematics, University College London London UK philip.pearce@ucl.ac.uk; Institute for the Physics of Living Systems, University College London London UK; Center for Systems Biology and Department of Pathology, Massachusetts General Hospital Boston Massachusetts USA; Department of Systems Biology, Harvard Medical School Boston Massachusetts USA; Wallace H. Coulter Department of Biomedical Engineering, Georgia Institute of Technology and Emory University Atlanta Georgia USA; Department of Pediatrics, Emory University School of Medicine Atlanta Georgia USA

## Abstract

Characterization of blood flow rheology in hematological disorders is critical for understanding disease pathophysiology. Existing methods to measure blood rheological parameters are limited in their physiological relevance, and there is a need for new tools that focus on the microcirculation and extract properties at finer resolution than overall flow resistance. Herein, we present a method that combines microfluidic systems and powerful object-tracking computational technologies with mathematical modeling to separate the red blood cell flow profile into a bulk component and a wall component. We use this framework to evaluate differential contributions of effective viscosity and wall friction to the overall resistance in blood from patients with sickle cell disease (SCD) under a range of oxygen tensions. Our results demonstrate that blood from patients with SCD exhibits elevated frictional and viscous resistances at all physiologic oxygen tensions. Additionally, the viscous resistance increases more rapidly than the frictional resistance as oxygen tension decreases, which may confound analyses that extract only flow velocities or overall flow resistances. Furthermore, we evaluate the impact of transfusion treatments on the components of the resistance, revealing patient variability in blood properties that may improve our understanding of the heterogeneity of clinical responses to such treatments. Overall, our system provides a new method to analyze patient-specific blood properties and can be applied to a wide range of hematological and vascular disorders.

## Abstract 2

We present a method that combines microfluidic systems and object-tracking computational technologies to evaluate the contributions of effective viscosity and wall friction to the overall resistance in blood from patients with sickle cell disease.

## Graphical abstract

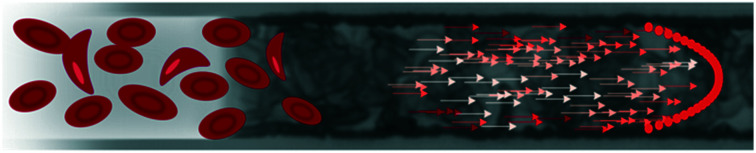

## Introduction

Sickle cell disease (SCD) is a hematological disorder that affects millions of people worldwide.^1^ Individuals suffering from this disease have a decreased life expectancy and a reduced quality of life owing to complications related to vascular pathologies such as vaso-occlusions, acute chest syndrome, and ischemic tissue and organ damage.^2^ SCD is caused by a genetic mutation resulting in a mutant hemoglobin molecule (sickle hemoglobin or HbSS), which can polymerize under deoxygenated conditions and induce changes in the biophysical properties of red blood cells (RBCs); these effects, consequently, lead to alterations in whole-blood rheology.^3,4^ Known links between blood rheology and the pathophysiology and progression of SCD include an increased likelihood of a vaso-occlusive crisis in blood that has a higher viscosity.^5,6^ Therefore, many clinical therapies aim to improve blood rheology, for example by introducing healthy RBCs through transfusion, or by introducing or promoting the expression of non-polymerizing hemoglobin using drugs or gene therapy. However, a clear identification of target rheological properties after such treatments has not been possible despite intensive efforts in the field.^7^ A particular difficulty is caused by the dependence of whole-blood properties on shear rate, hematocrit, and vessel size, all of which vary considerably throughout the vasculature.^8–11^ Patient-specific optimization of SCD treatment therefore requires a detailed understanding of how the rheological properties of the patient's blood depend on oxygen conditions throughout the vasculature, both before and after therapeutic intervention.

Our current understanding of whole-blood dynamics in SCD has been informed by numerous studies that have characterized macroscopic rheological properties using methodologies including specialized microfluidic devices and viscometers.^12–17^ Typically, such studies have measured only total flow resistances, which provide a crude estimate of the pressure required to drive flow through blood vessels. However, because blood in SCD patients (SCD blood) is a complex suspension, a more accurate characterization of its rheology should also capture non-Newtonian effects, such as shear thinning, and frictional effects arising from the interactions of RBCs with walls.^18–22^ Such frictional effects may be an important factor in chronic vascular endothelial inflammation and in precipitating vaso-occlusive crises. Improving our fundamental understanding of these various physical properties of blood, including how they depend on heterogeneity in RBC properties,^23–25^ will be a first step towards obtaining new patient-specific recommendations for the treatment of sickle cell disease.

Separating frictional and viscous resistance effects in multi-phase flows using rheometers requires careful calibration and measurement.^26–28^ Additionally, rheometers do not typically replicate microvasculature geometries or physiological oxygen tensions. To overcome these challenges, in this study we focus on time-resolved imaging of flow profiles. Existing velocimetry techniques that quantify features of flow at submicron resolution include particle image velocimetry (PIV) and laser Doppler velocimetry.^29–31^ Although the application of such techniques has improved our ability to quantify fluid properties and dynamics, some limitations restrict their application and usage. For example, in PIV, the use of artificial tracer particles can cause fluid distortion if the particles interfere with the flow dynamics. Moreover, for specialized particle suspensions, the resuspending mixture can prevent the native composition of the fluid of interest from being quantified. For example, stiff tracer particles will alter the rheology of a high volume fraction suspension of soft particles like blood.^32^ For laser doppler velocimetry and other velocimetry approaches, the composition of the imaging setup can require high-cost laser equipment, specialized optics, and carefully calibrated sensors and illumination placements, dissuading non-experts from utilizing these techniques.^33^ By contrast, the advancements in open-source or “off the shelf” computer vision over the past few years have been significant,^34,35^ and these methodologies are now widely used to track movement in a variety of applications using only natural contrast features as tracers. In this work, we exploit these widely available tools to visualize and quantify blood flow using a simple optical setup and without perturbing the blood's native properties.

The protocol that we introduce here employs a microfluidic platform, an open-source optical tracking algorithm, and theoretical modeling to characterize the frictional and macroscopic rheological properties of SCD blood and healthy blood. The microfluidic device provides control over relevant parameters such as oxygen concentration and pressure drop to mimic a range of conditions encountered in a physiological setting. This device also incorporates design features to obviate packing of red cells, which is a common technical challenge to studying impaired blood flow within microfluidic systems. The tracking method uses the Kanade–Lucas–Tomasi (KLT) feature-tracking algorithm, with the RBCs in the flow as natural tracer particles.^36^ RBC velocity values are then fit to a profile for a non-Newtonian fluid as a simple and computationally efficient theoretical model to extract fluid properties from their unique flow profiles. We demonstrate our protocol's utility by characterizing the macroscopic and frictional properties of healthy and SCD blood in a range of oxygen conditions, and by assessing how transfusion therapies affect blood properties. Overall, our protocol is simple-to-use and provides an efficient method for rheological characterization of complex suspensions.

## Methods

### Microfluidic device design and fabrication

**Fig. 1:**
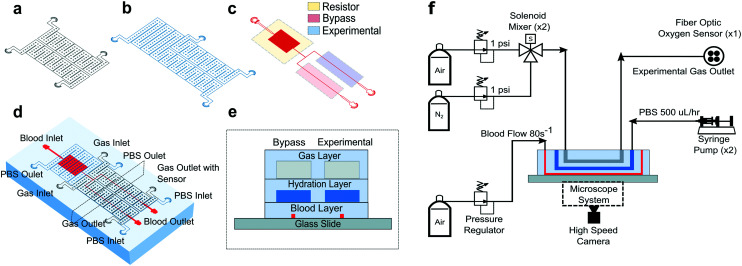
Device design and experimental setup. Our microfluidic device is a three-layer device with a (a) gas, (b) hydration, and (c) blood layer allowing blood flow shear rate and oxygen tension to be methodically controlled while time-resolved images are collected. (d) The multilayer device is fabricated using PDMS to PDMS plasma bonding techniques. The needle ports for fluid and gas inlets and outlets are punched and the PDMS device is subsequently bonded to glass (see Methods). (e) A cross sectional view of the device shows how the blood layer is separated from the hydration layer by 100 μm of PDMS (see Methods). The hydration layer is separated from the gas layer by 100 μm of PDMS using the same technique. The gas layer was fabricated to be 4 mm thick (images are not to scale). (f) For the experimental setup, a combination of hardware and controls systems are used to perfuse blood, maintain hydration, deliver different oxygen concentrations, and detect oxygen levels. An inverted, bright field microscope allows blood flow to be visualized and images to be acquired using a high-speed camera (representative figure, images are not drawn to scale).

All studies were conducted using a polydimethylsiloxane (PDMS) device and microfluidic system developed previously by our group.^14^ The microfluidic system allowed us to measure changes in SCD blood flow while controlling the oxygen concentration supplied to the blood. The device was a multilayer structure composed of a blood layer to mimic a blood vessel, a hydration layer to prevent blood dehydration and a gas layer used to regulate the oxygen partial pressure on the device through gas diffusive coupling (Fig. 1). The gas layer contained two, 150 μm tall channels. Each channel had an inlet port connected to a separate gas line, which allowed us to control the partial pressure of oxygen administered to two different regions of the blood channel (Fig. 1a and d). The hydration layer contained two, 100 μm tall channels covering the blood layer and was perfused with phosphate buffered saline (PBS) solution during experiments to prevent blood evaporation (Fig. 1b). The blood layer was manufactured as a rectangular channel with a geometric height of 15 μm and width of 20.6 ± 0.5 μm which corresponds to the cross-sectional area of arteriole/venule sized blood vessels (Fig. 1c). The blood channel was bifurcated into an experimental and bypass channel. During experiments, the bypass channel remained at 21% oxygen to prevent red blood cell packing while controlled hypoxia was delivered to the experimental channel. The bifurcation thus enhanced flow stability and helped maintain constant hematocrit levels. Additionally, the blood layer resistor section acted as a pressure dissipater to achieve a range of flow rates trackable by our computational algorithm.

Each layer was fabricated using soft lithography techniques from silicon wafer molds. The silicon wafer molds were fabricated using negative resist photolithography at the indicated feature geometries. The gas layer was fabricated using traditional PDMS (Sylgard 184, Dow Corning, USA) soft lithography to a targeted PDMS thickness of 4 mm. For the blood and hydration layer, a compression molding technique described previously^14^ was used to create a 100 μm thick layer of PDMS above the features. Using this technique, the blood/hydration interface and hydration/gas interface were separated by 100 μm thick PDMS membranes (Fig. 1e). The diffusive properties of the device and local oxygen concentration in the blood layer were previously validated using a COMSOL model and experimentation for the described geometries.^14^ Each layer was plasma bonded at 100 cc min^−1^ oxygen flow rate, 100 W power, and 60 seconds exposure time settings (PE-50, PlasmaEtch). The merged layers were then plasma bonded to a clean glass microscope slide using the same plasma settings to finalize device fabrication (Fig. 1e).

### Blood sample collection

Blood samples from healthy donors and donors with SCD were collected at the Massachusetts General Hospital under Institutional Review Board approved protocols (2006P000066) and Children's Hospital and Clinics of Minnesota under Institutional Review Board approved protocols (STUDY00003). All human subjects gave informed consent before participating in this study, or discarded specimens were used under an IRB-approved waiver of consent. Complete blood count (CBC) and high-performance liquid chromatography (HPLC) tests for all samples were conducted using a Sysmex XN-9000 automated analyzer and a Tosoh G7 column respectively to measure hematocrit, hemoglobin fractions, and other blood count indices for each sample. All blood samples tested were stored for a few hours and up to three days at 4 °C prior to testing. These storage conditions have been shown to not cause significant changes in rheological behavior of blood.^37,38^ For all measurements, sample plasma was removed by fractionating blood using a centrifuge at 400 g for 5 minutes and replaced with phosphate buffered saline solution. Hematocrit for all samples was fixed at 25% to limit hematocrit induced measurement variability *via* removal or addition of PBS to reach the target value. The hematocrit target was chosen based on average hematocrit for SCD patients.^39^

### Microfluidic system and testing setup

To characterize each blood sample, the microfluidic device described previously was visualized on a Zeiss Axio Vert microscope encased in a 37 °C environmental chamber. In the gas layer, oxygen at specified experimental concentrations was perfused using a solenoid valve gas mixer reported previously.^13^ The mixer works by combining air (21% O_2_, 5% CO_2_, balance N_2_) and 0% oxygen gas (5% CO_2_, balance N_2_) in a solenoid valve-controlled chamber to generate gas at user specified concentrations then perfused through the gas layers (Fig. 1f). Compressed air (21% O_2_, 5% CO_2_, balance N_2_) and compressed nitrogen (5% CO_2_, balance N_2_) were regulated to 1 psi (PRG200-25, omega) and their gas lines were connected to a manifold (Manifold, 3× HDI, 3-port, face mount, The Lee Co.) equipped with eight solenoid valves (3 port face mount solenoid valves, The Lee Co.). The oxygen concentration was set by the duty cycle of the solenoid valves operated by a control system. The control system included a control board (MCP23008 8-Channel 8 W Open Collector FET Driver I2C Shield with IoT Interface, NCD), an Arduino (Arduino Uno, Arduino) and custom MATLAB scripts. The mixing system could control up to eight independent gas chambers to allow for the decoupled gas section. During experiments, only two solenoid gas chambers were operated which allowed the bypass and experimental blood sections to be independently controlled. The oxygen tension at the device gas outlet was monitored for the duration of the experiment using a fiber optic oxygen sensor (NeoFox-GT, Ocean Optics). In the hydration channel, PBS was perfused using a 10 mL syringe pump at a rate of 500 μl h^−1^ (NE-500, New Era Pump Systems). Blood was perfused using an electronic pressure regulator (PCD-15PSIG, Alicat Scientific). Once a steady state average velocity of approximately 700 μm s^−1^ at 21% oxygen was achieved, the pressure was fixed for the duration of the experiment. Experimental blood pressure ranged from 1.0 to 1.5 PSI between experiments. The oxygen tension was cycled from 21% for 3 minutes down to an experimental oxygen tension (0–12%) for 5 minutes and back up to 21% for 3 minutes. This oxygen cycling was repeated for each experimental oxygen value. Video data of blood flowing in the microchannel was collected using a high-speed camera (GS3-U3-23S6M-C, FLIR) with a minimum frame rate of 70 frames per second (FPS) at max resolution, however acquisition of >400 FPS was typically achieved for our imaging conditions.

### Optical flow tracking analysis and validation

**Fig. 2:**
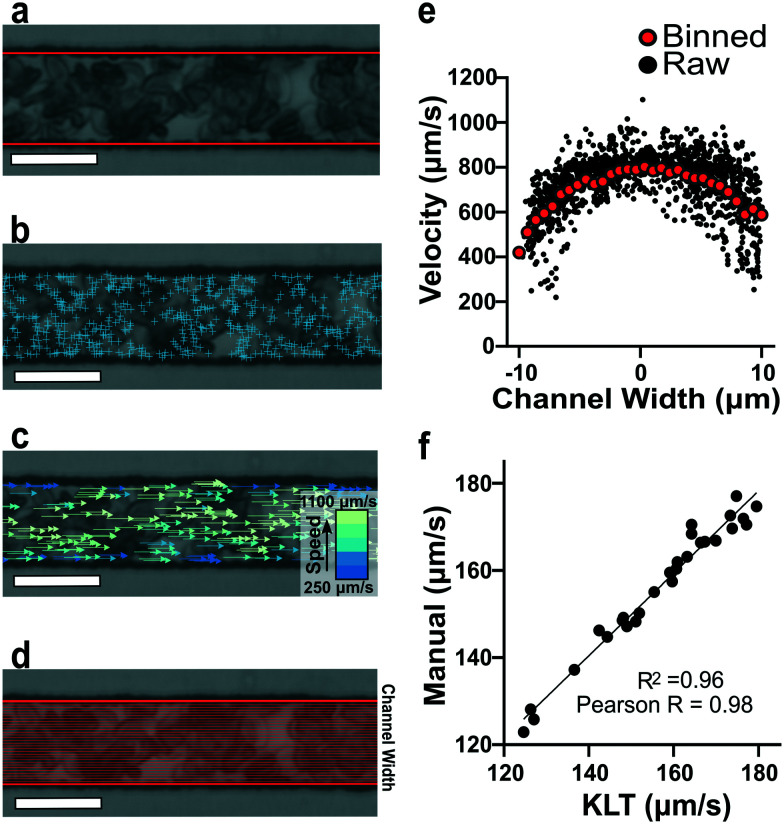
Quantification of red blood cell 1D velocity fields. (a) Bright field image of blood adjusted to 25% hematocrit (hct) inside the microfluidic device. The channel walls are automatically detected (red lines) and the feature-tracking algorithm is contained within this region of interest. (b) Implementation of the KLT algorithm using the MATLAB Computer Vision Toolbox and the MATLAB point tracker object identifies points of interest (blue ‘+’ symbols) in the initial frame of a 4-frame image stack. (c) Red blood cell velocities are calculated using the *x*-coordinate displacements computed from tracking the location of the points in sequential frames (see Methods). Velocities with the highest magnitude (green) are found in the center of the channel while velocities with the lowest magnitude (blue) are found near the channel walls. (d) Visual representation of the cross-sectional binning along the channel width for 30 bins. (e) Raw velocity data for each tracked point prior to binning as a function of channel width (black data points). Plot of binned velocity (30 bins, red data points). (f) Validation of RBC velocities determined manually and computationally (Pearson *r* = 0.98 and *r*_2_ = 0.96). (image scale bar = 20 μm).

**Fig. 3:**
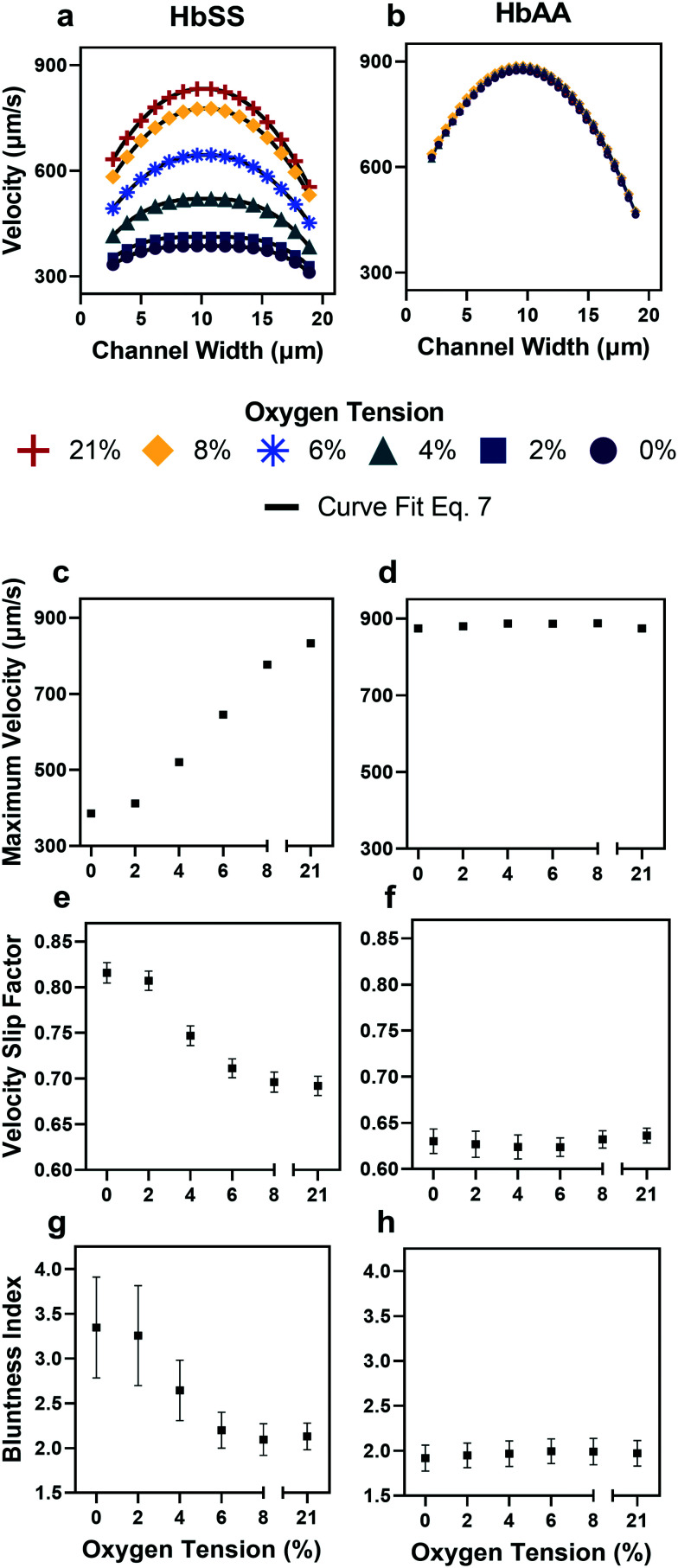
Qualitative and quantitative oxygen dependence of representative samples of healthy (HbAA) and SCD (HbSS) blood flow. All samples fixed to 25% hct (see Methods). (a) Raw velocity profiles for one SCD sample at a fixed pressure drop indicate that the velocity decreases with decreasing oxygen tension but also qualitatively the profiles seem to flatten. Black lines show the curve fit to eqn (7) for each oxygen tension. (b) Healthy donor sample profiles show no response to oxygen tension at fixed pressure drop. (c and d) Comparison of SCD and healthy sample maximum velocity. (e and f) Comparison of SCD and healthy sample slip velocity factor (*V*_wall_/*V*_max_). (g and h) Comparison of SCD and healthy sample bluntness index (b). Error bars represent sample-specific confidence interval for the fit of the velocity profile to eqn (7) at each oxygen tension.

To estimate red blood cell velocities using computer vision, the Kanade–Lucas–Tomasi (KLT) algorithm was used as a sparse feature-tracking method which can identify object features such as corners and edges based on image intensity gradients^40^ (Fig. 2). The KLT algorithm was incorporated into our custom image processing scripts using the MATLAB Computer Vision Toolbox and the vision.PointTracker object. Using this approach, the pixel coordinates of identified features were initialized and tracked among a set of consecutive frames and their positions saved. Experimental blood flow video data consisted of bright field 4-count image stacks captured and saved at a rate of >400 FPS every ∼1 second for the duration of the experiment (Fig. 2a). For each image stack, the KLT algorithm was applied, and features of interest were identified in the first frame (Fig. 2b). Using the KLT algorithm, the marked points were identified in the three consecutive frames and their position saved. To control for irregular data, points were validated between each frame using the forward-backward error approach^41^ and points that were lost from frame to frame or exited the channel region of interest were removed. Velocity field measurements (μm s^−1^) were computed using the frame-by-frame point displacements captured by the KLT method, frame rate acquisition speed, camera sensor size (5.86 μm per pixel) and microscope magnification (40×) (Fig. 2c). The velocity data was binned across the width of the channel (perpendicular to the direction of flow) and within each bin, an average velocity obtained (Fig. 2d). Using this process, a 1D velocity field was produced (Fig. 2e). A vertical bin size of 0.7 μm was used for all measurements because this size yielded >25 points per bin, which resulted in robust velocity estimates. Thus, the reported velocity profiles had 30 vertical bins with 0.7 μm resolution. The section of channel used for acquiring images was located at a distance approximately 75% the length of the experimental channel from the bifurcation point to allow for flow stabilization. The visualized section of channel had a region of interest of 21 by 150 μm to track at least 1000 points per image. These image acquisition criteria were used for each experiment. The data range was analyzed once the average velocity (determined using a real time tracking code) reached steady state at the specified experimental oxygen tension. The final velocity profiles, as seen in Fig. 3a and b, were computed from the average of 100 sets of 4-frame image stacks.

### Calculation of effective resistances

We separate the RBC flow rate *Q* in the experimental channel into a bulk component *Q*_bulk_ and a slip component *Q*_slip_ by conservation of mass.

1 *Q* = *Q*_bulk_ + *Q*_slip_

The slip component is calculated by multiplying the wall velocity (the velocity in the tracked bin nearest the wall) by the channel cross-sectional area (width *w* multiplied by height *h*). The bulk component is calculated by subtracting the wall velocity from the average velocity (obtained by integrating eqn (7)), then multiplying by the cross-sectional area.

2 *Q*_slip_ = *V*_wall_ × *w* × *h*

3 *Q*_bulk_ = (*V*_avg_ − *V*_wall_) × *w* × *h*

These calculations assume that the observed velocity profile approximately corresponds to a height-averaged velocity profile across the height of the device.^15^ Finally, effective resistances can be calculated from the flow rates by obtaining the pressure drop Δ*P* in the experimental channel *via* a linear network model of the device^42^ (see ESI† Text and Fig. S1a). The effective frictional resistance is calculated by dividing Δ*P* by *Q*_slip_, and the effective viscous resistance is calculated by dividing Δ*P* by *Q*_bulk_.

4 *R*_friction_ = Δ*P*/*Q*_slip_

5 *R*_viscous_ = Δ*P*/*Q*_bulk_

These equations imply that the frictional and viscous resistances act in parallel to determine the overall resistance (Fig. S1b†).6
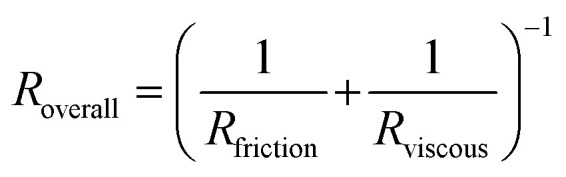


## Results

### Off-the-shelf computer vision algorithm provides accurate measurements of red blood cell velocities

To verify the accuracy of the KLT image tracking and binning algorithm, we compared the computational results to manually tracked videos collected under the same conditions as the image-tracking algorithm. For manual analysis, red blood cell (RBC) displacement was determined by recording the pixel location of their centroid position between two consecutive frames. Using known microscope magnification, pixel size, and video frame rate, a velocity in μm s^−1^ was determined for each cell and then averaged for the whole video. We compared the manual average velocity for 30 HbSS red blood cells at 21% oxygen to the average velocity acquired computationally to verify the computer vision algorithm accuracy. For the condition of 0.75 PSI pressure drop across the length of the device, manual tracking showed an average velocity of 156 ± 14.8 μm s^−1^ and computational tracking showed an average velocity of 157 ± 15.4 μm s^−1^. The resulting correlation coefficient of 0.98 confirms the accuracy of the computational velocity measurement (Fig. 2f).

### SCD blood flow profile metrics indicate a large RBC slip velocity and increased bluntness in response to hypoxia

The output of the flow tracking and binning algorithm is an empirical velocity profile that describes the average velocity of RBCs in different bins along the width of the channel (Fig. 2e). To characterize how the effect of oxygen tension on SCD blood differs from its effect on healthy blood, we measured such RBC velocities for representative HbSS (SCD) and HbAA (healthy) blood samples driven by a fixed pressure drop across the device (Fig. 3). We made three initial observations from these measurements. First, the velocity of HbSS blood appears to decrease as a function of oxygen tension, whereas the healthy sample showed no clear response to changes in oxygen tension (Fig. 3a and b). This observation is consistent with previous studies that have found the mean velocity of SCD blood to change in response to oxygen tension.^13,14,43^ The second observation is that there is a substantial apparent slip velocity at the wall for both the HbSS and HbAA blood samples, indicating that RBCs are able to slide along while close to the channel wall. A representative video to demonstrate this phenomenon is included (Video S1†). The existence of an apparent slip velocity is consistent with observations made of healthy blood flow in small vessels in mice.^44^ We found this slip velocity to depend strongly on oxygen for the SCD sample, but to be oxygen-independent for the healthy sample (Fig. 3a and b). Finally, we observed that the HbSS blood sample's velocity profile becomes more blunted as the oxygen tension decreases, whereas the shape of the HbAA blood sample remains constant over all oxygen (Fig. 3a and b).

To quantify these observations, which appear to demonstrate non-Newtonian behavior, we modeled the observed RBC velocity profile as an effective power-law fluid^45^ in which the velocity (*V*) depends on the distance from the channel center (*r*) as follows7
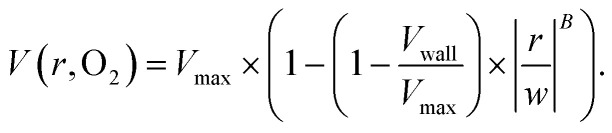
We emphasize here that our protocol tracks RBCs in the flow but does not track the suspending fluid – the connection between our simplified representation of the RBC dynamics (eqn (7)) and the velocity of the suspending fluid is not known. Our aim in applying eqn (7) is to quantify the salient features of the RBC flow *via* the fitted parameters *V*_wall_, *V*_max_ and *B*. We use empirical values for both *V* and *r* from the experimental setup and impose *w* as a fixed parameter based on measurements of the channel width. The effect of the cell free layer is accounted for by allowing a Navier slip condition *via* the parameter *V*_wall_ – this represents the RBC velocity near the wall and is consistent with a thin layer of suspending fluid between the RBCs and the wall, such that the RBCs closest to the wall have a non-zero velocity and the suspending fluid satisfies a no-slip condition at the wall (see Fig. S1b†). The fitted values of the parameters *V*_max_, *V*_wall_ and *B* quantify how changes in oxygen tension affect the RBC velocity profiles of our representative SCD and healthy blood samples (Fig. 3c–h). The bluntness index (*B*) describes the RBC velocity profile curvature; a bluntness index *B* = 2 implies a Newtonian RBC flow profile, and *B* > 2 implies a shear thinning RBC flow profile. We further define a slip velocity factor as the slip velocity divided by the maximum velocity, 
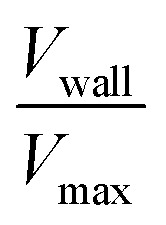
, as a measure of the importance of the effective slip velocity; a zero slip factor implies a no-slip condition, *V*_wall_ = 0, and a slip factor of 1 implies a plug flow, *V*_wall_ = *V*_max_ (Fig. 3e and f).

**Fig. 4:**
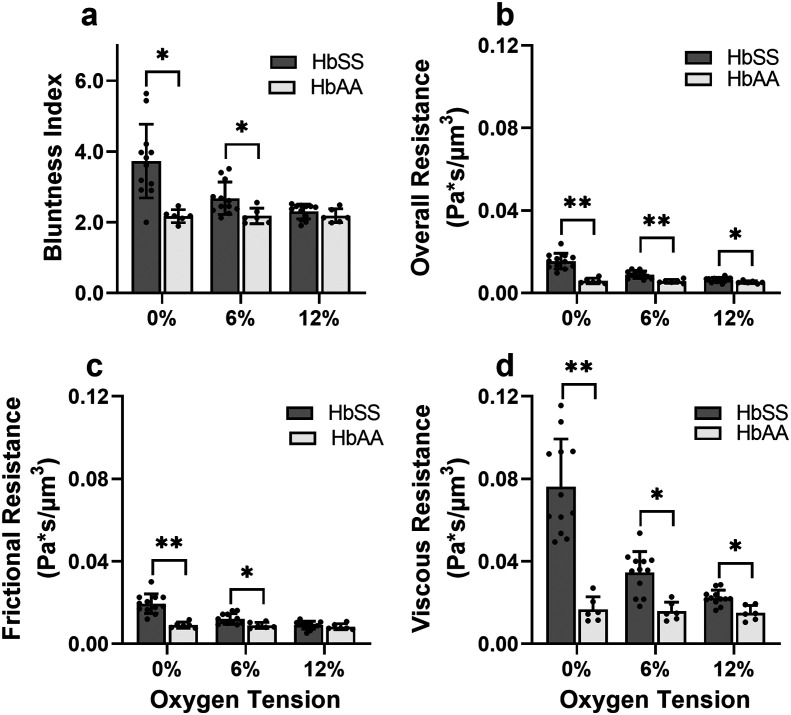
Evaluation of effective material properties of SCD blood across a range of samples. (a) Bluntness index, (b) overall resistance, (c) frictional resistance, and (d) viscous resistance. The differences in overall and viscous resistance between HbSS and HbAA samples are statistically significant at 12% oxygen tension. This finding shows a systemic effect that even at arterial level oxygen tensions (12%), HbSS sample blood flow properties are affected. Sample information: 12 HbSS samples, 6 HbAA samples. *p* values correspond to p* < 0.05 and p** < 0.001 using a Mann–Whitney *U* test in GraphPad Prism version 9.0.

In a representative sample of HbSS blood, we found that the slip velocity factor and bluntness index increase as oxygen tension is decreased below 8% (Fig. 3e and g). This indicates a flow with a flatter shape, in comparison to the more parabolic shape observed for fully oxygenated and healthy blood (Fig. 3a and b). Interestingly, we found the HbSS sample at high oxygenation (>6%) to have a higher slip factor and higher bluntness index than the healthy sample, suggesting differences in material properties and dynamics between healthy and SCD blood even at high oxygen tensions. Physical properties of the blood such as the effective viscosity and its dependence on the shear rate, as well as the interaction of the blood cells with the vessel wall, combine to determine the observed velocity profiles and therefore the fitted parameters *V*_max_, *B*, and *V*_wall_ . We further found that these trends hold across our cohort of 12 SCD blood samples and 6 healthy blood samples (Fig. 4a and S2a and b†).

### Effective flow resistances indicate sharp increases in effective viscosity and wall friction at lower oxygen tensions in SCD blood, which is consistently differentiable from healthy blood

To gain a deeper understanding of how hypoxia affects the properties of SCD blood, we wanted to extract macroscopic flow resistances that represent a qualitative description of the effect of oxygen tension on the overall material properties of the blood flow at the level of the entire channel. Therefore, we calculated an effective measure of the overall flow resistance by combining a network model to represent the bifurcations in the device (Fig. S1a†) with our fitted flow profile (eqn (7)); then, we separated this effective resistance into viscous and frictional components (eqn (4)–(6); see Methods). The effective viscous resistance is determined by the internal features of the flow such as RBC–RBC interactions, and the effective frictional resistance is determined by interactions between the RBCs and the wall. Our results show that even at physiological oxygen levels (12%), the overall and viscous resistances are statistically different between healthy and SCD samples (12 HbSS blood samples and 6 HbAA blood samples; see Fig. 4b and c). Furthermore, our analysis across a range of samples suggests that the overall, frictional, and viscous resistances increase sharply in HbSS samples as oxygen tension is reduced from 6% to 0% (Fig. 4b–d). Interestingly, our results suggest that as oxygen tension decreases, maintaining blood flow becomes increasingly dependent on frictional effects; this is because the viscous resistance becomes much larger than the frictional resistance at low oxygen tensions, and the two resistances effectively act in parallel (see eqn (6) and Fig. S1b†).

### Flow profile quantitative metrics allow the evaluation of transfusion therapies

Recent work suggests that populations of RBCs in SCD blood can show significant heterogeneity in hemoglobin polymer fraction at reduced oxygen tension.^23^ RBC heterogeneity is also introduced by blood transfusions, which are a common treatment for SCD patients that work by diluting the SCD red cells with healthy donor cells. Clinically, a typical target value for a red cell exchange procedure (erythrocytapheresis) is a 30% HbS fraction; however, it is not known how this target value translates to the rheological properties of blood and the risk of vaso-occlusive crisis in individual patients. To investigate how heterogeneity in RBC properties affects the macroscopic properties of blood in different patients, we evaluated *in vitro* blood transfusion samples by mixing type-matched blood with HbSS : HbAA red blood cell ratios of 0 : 100, 30 : 70, 70 : 30, and 100 : 0. Exact HbS fractions were calculated *post hoc* once hemoglobin variant was available, and all information about the blood samples can be found in the Table S1.† We extracted viscous and frictional resistances from these samples under the same conditions as our previous experiments across three oxygen tensions (0%, 6%, and 12%).

**Fig. 5:**
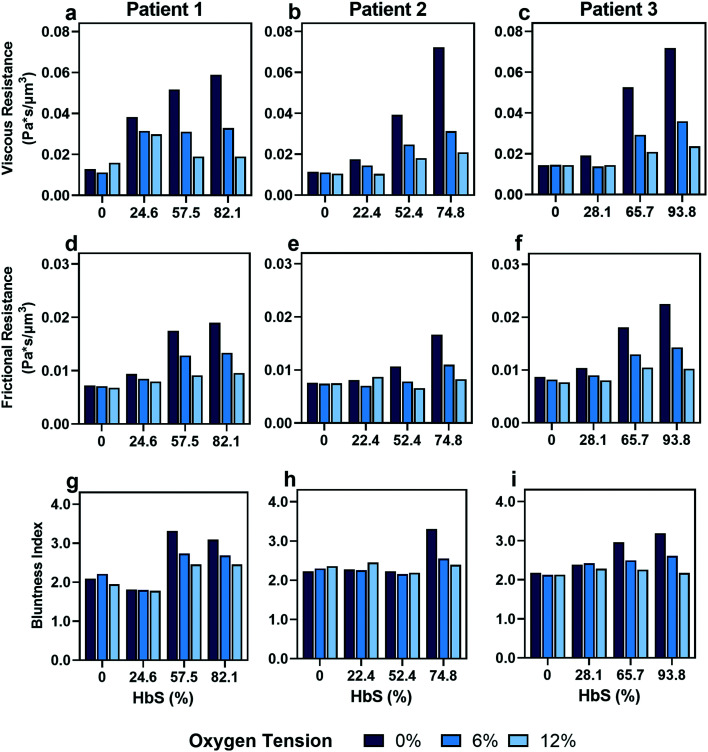
*In vitro* transfusion therapy experiment reveals increasing healthy to SCD blood ratios (indicated by decreasing HbS concentrations) leads to a loss of blood rheological dependence on oxygen tension, with heterogeneity in patient responses to transfusion. (a–c) Viscous resistance, (d–f) frictional resistance, and (g–i) bluntness index results for 3 patients at 3 different oxygen tensions (0%, 6%, and 12%). Patient 1 (column 1), patient 2 (column 2) and patient 3 (column 3) HbS concentrations before transfusion were 82.1%, 74.8% and 93.8%, respectively, and correspond to a SCD : healthy blood ratio of 100 : 0.

As expected, we found that decreasing the proportion of SCD cells reduces the effect of oxygen tension on both resistances that we calculated (Fig. 5). Despite a consistency in the overall trends, we observed variability in the effect of transfusions between parameters and between patients. For example, Patient 2 and 3 showed improvements in viscous resistance at each oxygen tension when the HbS concentration decreased from 52% to 22% and 65% to 28%, respectively, as suggested by the convergence of the viscous resistance values to those of donor blood (0% HbS). In contrast, patient 1 did not show a comparable improvement to the level of donor blood when the HbS fraction decreased from approximately 57% to 24%, and showed such improvement only when the HbS fraction was decreased to 0% (Fig. 5a–c). Furthermore, we found that Patient 2 showed a large improvement in frictional resistance between 74% and 52% Hbs (Fig. 5e), in contrast to patients 1 and 3, whose samples did not show drastic improvements in frictional resistance until HbS was reduced to around 24% and 28%, respectively (Fig. 5d and f). Patient 2 showed minimal changes in shear thinning properties with respect to oxygen at an Hbs fraction of 50% (Fig. 5h) but exhibited noticeable changes in viscous and frictional resistance (Fig. 5b and e). Additionally, two more patient transfusions were included and the results are in agreement with the heterogeneity observed regarding the effect of transfusions on rheological properties (Fig. S3†). Although further analysis is needed to understand the basis for the observed heterogeneity, these results provide evidence that some patients may benefit from the effects of transfusions at HbS fractions greater than 30% while for others, the targeted 30% HbS may not sufficient.

## Discussion

This study demonstrates how the constituents of a complex suspension in a microfluidic system can be used as tracer particles for a widely available image tracking algorithm to compute high resolution velocity fields (Fig. 2). We have applied this protocol to extract measures of the physical properties of blood in health and disease from the dynamics of red blood cells (RBCs; see Fig. 3). We have analyzed blood samples from a small cohort of patients with sickle cell disease (SCD) and compared the results to blood from patients without SCD. Our results demonstrate that the overall effective flow resistance of the RBC flow profile can be separated into viscous and frictional components, which act in parallel to determine the overall resistance (eqn (6)). Because of this, analyses that extract only flow velocities may be confounded – for example, an increase in viscous resistance would be expected to lead to an increase in slip velocity even if the pressure drop and frictional resistance remained the same. We note that the effective frictional resistance of the RBC profile is finite because of the cell-free layer, which allows the RBCs nearest the wall to continue to flow, despite the fact that the suspending fluid satisfies a no-slip condition (Fig. S1b†) – we expect that a higher effective frictional resistance corresponds to a thinner cell-free layer. We found that the two components of the overall effective resistance increase differentially in samples from SCD patients as oxygen tension is reduced (Fig. 4). Interestingly, we found the viscous resistance of SCD blood samples to be so large at 0% oxygen that the overall resistance is essentially determined by the frictional resistance.

We note that the effective resistances apply to the RBC velocity profile, which is measured by our algorithm. We have made significant simplifications and assumptions about the RBC flow to extract the resistances from the measured profile. By assuming that the RBC velocity profile satisfies eqn (7), we have assumed that the RBCs collectively behave as a single power-law fluid. Furthermore, we do not extract information about the suspending fluid, because it is not tracked by our imaging protocol. To understand the cell-level determinants of our observations, and to infer the dynamics of the suspending fluid, will require multi-scale models that resolve the dynamics of individual cells in whole blood. We note also that more detailed continuum models than eqn (7) could be applied to the RBC profile – for example, although our results are consistent with the shear thinning nature of blood, the empirical velocity profile at very low oxygen tensions is also consistent with a measurable yield stress^46^ which is not captured in our model. However, the simple nature of the model has allowed us to extract clear physical features of the blood in SCD that merit further investigation.

There are various clinical implications of this work. A major finding of this study is that the effective flow resistance of SCD blood is significantly higher than that of healthy blood even under normoxic conditions. Previous studies showed that SCD whole blood had higher oxygenated viscosities than healthy blood and concluded that plasma components were the primary contributing factor.^47^ Our results, which isolate the RBC contribution to blood viscosity, show that SCD RBCs have a significant contribution as well. Increased flow resistance under normoxic conditions increases the chronic risk for vascular pathologies throughout the vasculature, including in the arteries and in high oxygen tissues such as the brain. Our finding that the effective flow resistance of SCD blood is increased relative to healthy blood under all oxygen tensions is consistent with the clinical evidence of chronically elevated cardiac afterload, including concentric hypertrophy, diastolic dysfunction, and systolic hypertension in the peripheral arterial system of individuals with SCD.^48,49^ Another clinically relevant result of our study is that the effective frictional resistance increases in SCD blood across all oxygen tensions. This result suggest that sickle RBCs interact more than healthy RBCs with the vessel wall under hypoxic conditions, providing a potential biophysical mechanism for chronic endothelial inflammation beyond the biochemical mechanisms previously discovered^50^ – even in the absence of free heme or other biochemical triggers, altered blood properties may cause chronic injury to the endothelium throughout the vasculature. Increased RBC-endothelium interactions also increase the likelihood for endothelial adhesion and the formation of vaso-occlusions. Together, our findings highlight potential biophysical underpinnings of chronic and acute pathologies observed throughout the circulation in SCD, including vaso-occlusion, endothelial inflammation, acute chest syndrome, aneurysm, and stroke.^51^

Our *ex vivo* transfusion studies confirm that individual SCD patients have variable responses to transfusion. We observed variability in the effective resistances of blood between patients, in agreement with previous studies linking variations in patient hematologic profiles, such as differences in quantities of sickle hemoglobin (HbS) and fetal hemoglobin (HbF), to variations in blood flow properties.^15^ Our results suggest that patient-specific treatments may be more effective than population-averaged treatment strategies in ameliorating symptoms while reducing treatment-related complications. For example, further evaluation of how these resistances connect to vaso-occlusive risk in a range of patients may help with the identification of targeted values that conform to patients' specific needs, preventing common transfusion issues such as alloimmunization and iron overload. Moreover, these findings provide the opportunity to tailor combination therapies for individual patients. For example, patients with high frictional resistance that is not abated by transfusion may benefit from combination with therapies such as Crizanlizumab, which inhibits adhesion of RBCs to the endothelium.^52^ The information required to inform such targeted treaments is enabled only by using an approach that decouples unique biophysical mechanisms, such as the one described here.

In addition to elucidating mechanisms of SCD pathophysiology, this study validates our method, which uses a basic light microscope, an inexpensive machine vision camera, and an off-the-shelf computer vision algorithm to compute high-resolution velocity fields in a complex fluid. This obviates the need for complex optical setups and lighting and allows for application of this label-free PIV method in a wide range of microfluidic applications. Like any PIV method, the KLT algorithm requires trackable features as fluid tracers. Here, we used the RBCs, which were the feature of interest for our measurements. A major advantage of using the RBCs is that we explicitly measure the velocity of one of the fluid phases, whereas artificial tracers would not directly measure either the continuous phase or the RBCs, making interpretation more difficult. Moreover, we can measure the blood in its native composition without perturbation from artificial tracers. However, in measurements without natural tracers, inexpensive tracers such as unlabeled polystyrene beads could be used without limiting the wide applicability of the method.

## Conclusion

We have presented a new method that combines microfluidics, optical tracking, and modeling approaches to quantify the physical characteristics of complex suspensions from the dynamics of their constituents, without the need for artificial tracer particles. In this study, we have applied our method to compare the macroscopic properties of blood from sickle cell disease (SCD) patients with the properties of healthy blood and of simulated transfusions. We have found that effective measures of the frictional and viscous flow resistance increase differentially in blood from SCD patients in reduced oxygen tensions. Furthermore, we have found that the effective viscous resistance is higher in SCD blood than in healthy blood even in highly oxygenated conditions. These results highlight biophysical mechanisms that could contribute to chronic and acute pathologies in SCD patients. We anticipate that, in the future, it will be possible to use a system such as ours to perform cheap and efficient patient-specific analysis of SCD blood. Combining our results with clinical outcomes will allow such analyses to determine vaso-occlusive risk and predict optimal transfusion ratios and treatment regimens that reduce pathology in SCD.

## Conflicts of interest

There are no conflicts to declare.

## Supplementary Material

LC-022-D1LC01133B-s001

LC-022-D1LC01133B-s002

LC-022-D1LC01133B-s003
