# Supplementary material for: Feature tracking microfluidic analysis reveals differential roles of viscosity and friction in sickle cell blood
Source: Lab Chip. 2022 Mar 16;22(8):1565–75. doi: 10.1039/d1lc01133b (PMC9004467; doi:10.1039/d1lc01133b)
Supplement: LC-022-D1LC01133B-s001 [file LC-022-D1LC01133B-s001.pdf]

Electronic Supplementary Material (ESI) for Lab on a Chip.
This journal is © The Royal Society of Chemistry 2022

Supplementary Information: Feature tracking microfluidic analysis reveals differential roles of viscosity and friction in sickle cell blood

Hannah M. Szafraniec**, Jose M. Valdez**, Elizabeth Iffrig, Wilbur Lam, John M. Higgins, Philip Pearce*, David K. Wood*

Calculation of pressure drop across bypass channel

In the microfluidic device, the "inflow" channel splits into two separate channels – the "experimental" channel and the "bypass" channel (see Fig. 1a in the main paper). We aim to calculate the pressure drop across the experimental channel. To this end, we first apply Poiseuille's law in each channel

$$1)\quad \Delta P_{bypass} = Q_{bypass} * R_{bypass}$$

$$2)\quad \Delta P_{experimental} = \Delta P_{bypass} = Q_{experimental} * R_{experimental}$$

$$3)\quad \Delta P_{inflow} = Q_{inflow} * R_{inflow}$$

where ΔP is the pressure drop, Q is the flow rate, R is the resistance, and the channel is denoted by the subscripts. The overall pressure drop across the device is a continuous function, so that

$$4)\quad \Delta P_{total} = \Delta P_{inflow} + \Delta P_{bypass}$$

By conservation of mass, we have

$$5)\quad Q_{total} = Q_{bypass} + Q_{experimental}$$

In each experiment, the resistances of the inflow and bypass channel are known, the total pressure drop is imposed, and the flow rate in the experimental channel is measured. By combining Eq. 1 through 5, we can solve for the pressure drop across the experimental channel in terms of these known variables to obtain

$$6)\quad \Delta P_{experimental} = \frac{\Delta P_{total} - R_{inflow} * Q_{experimental}}{R_{inflow}/R_{bypass} + 1}$$

The calculation requires the effective resistances of the inflow and bypass channels, which are fixed because the oxygen tension is not varied in those channels. To obtain these effective resistances, we combine measurements of the flow rate in the experimental channel at full oxygenation (>12%, when the resistances of the experimental and bypass channel are the same) with available formulas for the hydraulic resistances of rectangular channels[1].

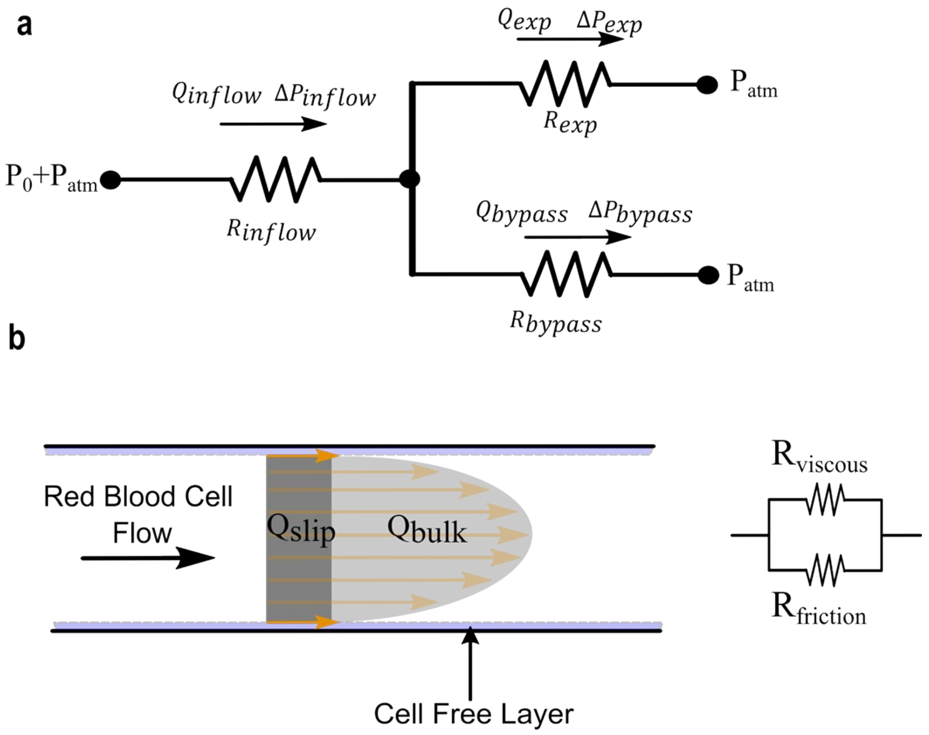

Figure S1: (a) Resistor network for calculation of pressure drop across bypass channel. (b) Visual representation of the cell free layer and separation of red blood cell flow into slip and bulk components. The overall resistance in the channel can be separated into a frictional component and viscous component that act in parallel (see Methods in the main paper).

Video S1: Representative video of HbSS blood flow in our microfluidic device at 21% oxygen and 1.5psi with an average velocity of 700 µm/s. The video demonstrates the ability for red blood cells to slide along near the walls of the device.

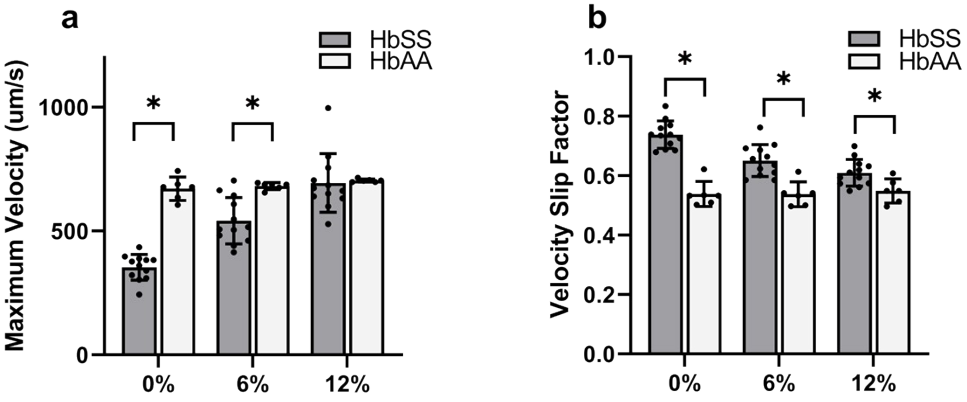

Figure S2: Fitted parameters for HbSS blood samples (n=12) and HbAA blood samples (n=6). The (a) maximum velocity and the (b) velocity slip factor both show oxygen dependent effects for the HbSS samples. P values correspond to $p^* < 0.05$ using a Mann-Whitney U test.

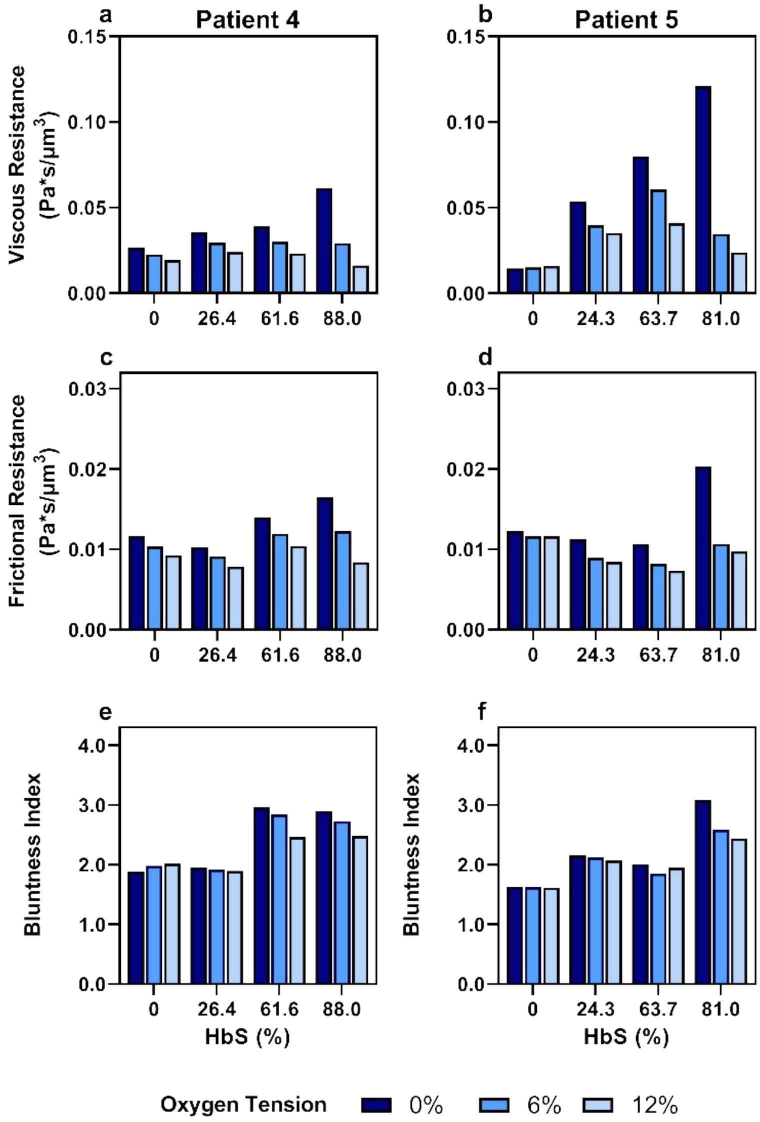

Figure S3: In-vitro transfusion therapy experiment for two additional patients. (a-b) Viscous resistance, (c-d) frictional resistance, and (e-f) bluntness index results for two patients at three different oxygen tensions (0%, 6%, and 12%). Patient 4 (column 1) and Patient 5 (column 2) HbS concentrations before transfusion were 88.0% and 81.0%, respectively.

	Sample ID	HbS	HbF	HbA	HbA2	HCT	MCV	MCHC	WBC	Transfusions	Hydroxyurea	Genotype	Gender
	1064	72.60%	10.50%	11.50%	5.30%	19.8	109	34.8	8.22	unknown	YES	HbSS	F
	1044	77.90%	5.90%	9.30%	6.90%	26.5	86.9	32.8	14.2	unknown	YES	HbSS	M
	1162	85.10%	0.00%	9.30%	5.70%	23.8	67.2	33.2	16.9	unknown	unknown	HbSS	F
	1065	72.60%	11.00%	11.30%	5.10%	18.8	110	35.1	7.37	YES	YES	HbSS	F
	1163	88.60%	4.30%	0.00%	7.10%	26.3	82.4	32.3	16	unknown	YES	HbSS	M
	845	86.10%	5.30%	0.00%	8.70%	24.4	77.7	36.9	4.41	unknown	YES	HbSS	F
	851	85.40%	10.20%	0.00%	4.40%	24.6	91.1	35.8	6.94	Unknown	YES	HbSS	M
	895	92.80%	0.00%	0.00%	7.20%	29.3	88	32.4	10.9	NO	NO	HbSS	F
Transfusion Patient 1	860	82.10%	13.50%	0.00%	4.40%	20	98	35.5	7.55	YES	YES	HbSS	F
Transfusion Patient 2	837	74.80%	7.30%	13.50%	4.40%	27.1	111	34.7	9.69	unknown	YES	HbSS	M
Transfusion Patient 3	844	93.80%	0.00%	0.00%	6.20%	21	65.4	31.9	12.3	unknown	unknown	HbSS	F
Transfusion Patient 4	859	88.80%	6.30%	0.00%	4.90%	19.7	115	36	11.9	unknown	YES	HbSS	M
Transfusion Patient 5	25	81.00%	15.10%	0.00%	2.90%	22.7	89	33.5	6.9	NO	YES	HbSS	M

Table S1: Patient hematological profiles for 12 HbSS patient samples and one additional HbSS sample for transfusion experiments (patient 5).

1. Bruus, H. Theoretical microfluidics. *Choice Reviews Online* **45**, 45-5602-45–5602 (2008).
