# Supplementary material for: Feature tracking microfluidic analysis reveals differential roles of viscosity and friction in sickle cell blood
Source: Lab Chip. 2022 Mar 16;22(8):1565–75. doi: 10.1039/d1lc01133b (PMC9004467; doi:10.1039/d1lc01133b)
Supplement: LC-022-D1LC01133B-s002 [file LC-022-D1LC01133B-s002.pdf]

Electronic Supplementary Material (ESI) for Lab on a Chip.
This journal is © The Royal Society of Chemistry 2022
